# Sugar Accumulation in Leaves of Arabidopsis *sweet11/sweet12* Double Mutants Enhances Priming of the Salicylic Acid-Mediated Defense Response

**DOI:** 10.3389/fpls.2017.01378

**Published:** 2017-08-08

**Authors:** Pierre Gebauer, Martin Korn, Timo Engelsdorf, Uwe Sonnewald, Christian Koch, Lars M. Voll

**Affiliations:** Division of Biochemistry, Friedrich-Alexander-Universität Erlangen-Nürnberg Erlangen, Germany

**Keywords:** pathogen nutrition, SWEET, Arabidopsis, sugar transport, phloem loading, carbon metabolism, salicylic acid, defense priming

## Abstract

In compatible interactions, biotrophic microbial phytopathogens rely on the supply of assimilates by the colonized host tissue. It has been found in rice that phloem localized SWEET sucrose transporters can be reprogrammed by bacterial effectors to establish compatibility. We observed that *sweet11*/*sweet12* double mutants, but not single mutants, exhibited increased resistance toward the fungal hemibiotroph *Colletotrichum higginsianum* (*Ch*), both in the biotrophic and the necrotrophic colonization phase. We therefore investigated if the phloem localized transporters *At*SWEET11 and *At*SWEET12 represent additive susceptibility factors in the interaction of Arabidopsis with *Ch*. *At*SWEET12-YFP fusion protein driven by the endogenous promoter strongly accumulated at *Ch* infection sites and in the vasculature upon challenge with *Ch*. However, susceptibility of *sweet12* single mutants to *Ch* was comparable to wild type, indicating that the accumulation of *At*SWEET12 at *Ch* infection sites does not play a major role for compatibility. *At*SWEET12-YFP reporter protein was not detectable at the plant–pathogen interface, suggesting that *AtSWEET12* is not targeted by *Ch* effectors. *At*SWEET11-YFP accumulation in *pAtSWEET11:AtSWEET11-YFP* plants were similar in *Ch* infected and mock control leaves. A close inspection of major carbohydrate metabolism in non-infected control plants revealed that soluble sugar and starch content were substantially elevated in *sweet11*/*sweet12* double mutants during the entire diurnal cycle, that diurnal soluble sugar turnover was increased more than twofold in *sweet11*/*sweet12*, and that accumulation of free hexoses and sucrose was strongly expedited in double mutant leaves compared to wild type and both single mutants during the course of *Ch* infection. After 2 days of treatment, free and conjugated SA levels were significantly increased in infected and mock control leaves of *sweet11*/*sweet12* relative to all other genotypes, respectively. Induced genes in mock treated *sweet11*/*sweet12* leaves were highly significantly enriched for several GO terms associated with SA signaling and response compared to mock treated wild-type leaves, indicating sugar-mediated priming of the SA pathway in the double mutant. Infection assays with salicylic acid deficient *sweet11*/*sweet12/sid2* triple mutants demonstrated that reduced susceptibility observed in *sweet11*/*sweet12* was entirely dependent on the SA pathway. We suggest a model how defects in phloem loading of sucrose can influence SA priming and hence, compatibility.

## Introduction

Prokaryotic and eukaryotic plant pathogens drive their own metabolism by diverting organic and inorganic solutes from colonized host tissue. Nutrient acquisition from host cells and their efficient uptake is crucial for the successful establishment of phytopathogens *in planta* (reviewed by Divon and Fluhr, 2007).

Plant pathogens have evolved different strategies to acquire organic nutrients from their host plants. After penetration by wounds or natural openings, necrotrophic pathogens rapidly kill the plant tissue by the secretion of highly efficient toxins and/or cell wall degrading enzymes (as reviewed by van Kan, 2006) and can utilize simple organic carbon and nitrogen sources as well as building blocks liberated by hydrolysis of complex polymers. In contrast, biotrophic pathogens strictly rely on the supply of organic carbon and nitrogen metabolites by living host tissue (Divon and Fluhr, 2007). Due to a substantial diversion of C and N assimilates, infected leaf tissues are transformed into strong local sinks at the infection site (e.g., Biemelt and Sonnewald, 2006).

It has been shown that fungal biotrophs require metabolite transporters for the uptake of host assimilates, but it is yet unclear, which host functions mediate the export of carbon and nitrogen assimilates into the apoplasmic interface between host and pathogen. Seminal work on the bean rust fungus *Uromyces fabae* lead to the identification of a proton-coupled hexose transporter and two proton-coupled amino acid transporters that were induced in fungal haustoria (Hahn et al., 1997; Voegele et al., 2001; Struck et al., 2002, 2004). A secreted fungal invertase was found to be induced in rust infected leaves (Voegele et al., 2006), which is thought to ensure the supply of hexoses to the fungus by the cleavage of sucrose in the extrahaustorial matrix. Many fungal and bacterial phytopathogens studied to date either produce secreted invertases or induce host-derived cell wall invertases to secure the provision of hexoses as carbon source (Billett et al., 1977; Heisterüber et al., 1994; Chou et al., 2000; Fotopoulos et al., 2003; Swarbrick et al., 2006; Voegele et al., 2006; Horst et al., 2008; Hayes et al., 2010). Direct uptake of sucrose, as by the proton-coupled high-affinity *Ustilago maydis* sucrose transporter Srt1 (Wahl et al., 2010), remains an exception. In some cases, sufficient activity of extracellular invertases (Siemens et al., 2011) or direct sucrose uptake by sucrose transporters (Wahl et al., 2010) are indispensable for full virulence, indicating that sucrose is the major carbon source of these pathogens *in planta*. In contrast, loss of citrate uptake, but not the loss of sucrose uptake, compromised *in planta* proliferation of *Xanthomonas campestris* pv. *vesicatoria* (*Xcv*) (Tamir-Ariel et al., 2011), demonstrating that the organic acid citrate is the preferred carbon source for this bacterial phytopathogen. It also needs to be noted that favorable organic nitrogen sources like glutamine (Clark and Hall, 1998; Horst et al., 2010), asparagine (Horst et al., 2010) or GABA (Solomon and Oliver, 2001) provide excess carbon along with organic N to fungal pathogens.

Neutral sugars represent an important carbon source for most biotrophs studied to date. The induction of bidirectional sugar uniporters of the SWEET (SUGARS WILL EVENTUALLY BE EXPORTED TRANSPORTER) family by TAL (transcriptional activator like)-effectors was shown to be required for virulence of the bacterial rice pathogen *Xanthomonas oryzae* pv. *oryzae* (*Xoo*) (Chen et al., 2010). Four different bacterial TAL effectors are known to induce the rice sugar transporters *Os*SWEET11 or *Os*SWEET14 (Chu et al., 2006; Yang et al., 2006; Antony et al., 2010; Chen et al., 2010; Yuan et al., 2011). In compatible interactions, at least one of these two target SWEET genes is induced by *Xoo*. Recessive bacterial blight resistance alleles of both *SWEET* transporter genes were described, in which mutated TAL effector binding sites in the promoter regions of the respective SWEET genes prevent reprogramming by the pathogen (Chu et al., 2006; Yang et al., 2006; Chen et al., 2010; Liu et al., 2011; Yu et al., 2011; Yuan et al., 2011). This suggests that enhanced sucrose export by SWEET transporters may be essential for nutrition of *Xoo*, which proliferates in the xylem, an environment that is typically poor in organic carbon sources. With the help of artificially designed TAL effectors, (Streubel et al., 2013) demonstrated that only the induction of clade III SWEET transporters is sufficient to support virulence of *Xoo*. Interestingly, all characterized clade III SWEET transporters were shown to transport sucrose (Chen et al., 2012). Recently, the *Xanthomonas axonopodis* pv. *manihotis* (*Xam*) TAL effector TAL20 was identified to target *MeSWEET10a*, a clade III hexose and sucrose transporter, in cassava (Cohn et al., 2014). However, TAL20 deletion strains only exhibited a moderately reduced virulence, while *in planta* proliferation was not impaired (Cohn et al., 2014), indicating that induction of *MeSWEET10a* by *Xam* is dispensable for pathogenicity.

Taken together, this may either indicate that substrate specificity of the induced SWEET transporters is important for the establishment of compatibility in the individual pathosystem or that SWEET transporters are not the major route of carbon supply for all pathogens. Among other *SWEET* genes, *AtSWEET11* and *AtSWEET12* were shown to be induced during infection of Arabidopsis leaves with biotrophic, hemibiotrophic and necrotrophic pathogens, suggesting that reprogramming of *SWEET* transporters may represent a common strategy for nutrient acquisition of Arabidopsis pathogens (Chen et al., 2010). Since *AtSWEET11* and *AtSWEET12* exert redundant functions in phloem loading with sucrose and are the most abundant clade III transporters in leaves (Chen et al., 2012), we investigated the role of Arabidopsis *AtSWEET11* and *AtSWEET12* during the interaction with the adapted fungal hemibiotroph *Colletotrichum higginsianum* (*Ch*), which had not been studied by Chen et al. (2012).

*Colletotrichum higginsianum* forms haploid conidiospores, which land on the plant surface and differentiate specialized penetration organs, so called appressoria. Penetration pegs emerge at the bottom of the appressoria and breach the underlying epidermal cell walls predominantly by mechanical force (Bechinger, 1999; Deising et al., 2000). To build up sufficient turgor pressure for this process, *Colletotrichum* appressoria accumulate sugar alcohols and require osmosis-driven water supply from the outside, e.g., by rain drops (Bechinger, 1999; Deising et al., 2000). In the first penetrated epidermis cell, *Ch* establishes itself as a biotroph within 36 h post inoculation by forming a bulbous infection vesicle that subsequently produces lobed biotrophic primary hyphae (O’Connell et al., 2004). Recent data indicate that this stage may rather serve defense suppression than nutrient uptake: stage specific RNAseq analysis could not identify transporters for major organic carbon and nitrogen that were strongly induced in the biotrophic phase (O’Connell et al., 2012). Furthermore, an almost quantitative depletion of soluble sugars from infected host tissue during biotrophic colonization did not result in attenuated growth of *C. higginsianum*, but rather lead to increased host susceptibility (Engelsdorf et al., 2013). Hyphal morphology and fungal lifestyle change at around 72 h post inoculation, when neighboring cells are colonized by rapidly growing necrotrophic secondary hyphae (SH) of *Ch*, which leads to visible necrotic lesions on infected leaves that contain acervuli with newly formed conidiospores.

The data presented here suggest that the local induction of *AtSWEET12* at infection sites does not support fungal nutrition and proliferation, while *AtSWEET11* is not locally induced. The absence of AtSWEET12 accumulation at the direct plant–fungal interface makes it unlikely that *AtSWEET12* expression is locally induced by fungal effectors. Our results further demonstrate that reduced susceptibility of Arabidopsis *sweet11/sweet12* double mutants toward *Ch* is caused by sugar-induced priming of the SA pathway.

## Materials and Methods

### Plant Lines, Fungal and Bacterial Strains and Growth Conditions

Arabidopsis plants were grown in short day (8 h light/16 h dark), 12 h light/12 h dark and long days (16 h light/8 h dark) as described in Engelsdorf et al. (2013). All mutants, *At*SWEET11-GFP and *At*SWEET12-GFP reporter plants used in this study are described in Chen et al. (2010, 2012), except for *sid2-3* (SALK_042603) that is described in Gross et al. (2006). p*AtSWEET11*:*AtSWEET11*-YFP and p*AtSWEET12*:*AtSWEET1*2-YFP reporter constructs were made by cloning p*AtSWEET11*:*AtSWEET11* and p*AtSWEET12*:*AtSWEET1*2, respectively, from the donor vector pDONR221-f1 (Chen et al., 2012) into the pEG-TW Gateway vector (Chen et al., 2012; Fan et al., 2014) by LR clonase. Transgenic plants were produced as described previously (Chen et al., 2012) and were a generous gift of Li-Qing Chen and Wolf Frommer (Carnegie Institution for Science, Stanford, CA, United States). All mutants and transformants were in the Col-0 background.

*Colletotrichum higginsianum* wild type isolate MAFF 305635 (Ministry of Agriculture, Forestry and Fisheries, Japan) and CIH1-mCherry overexpressors were used. The CIH1-mCherry expressing strain was generated by Agrobacterium mediated transformation of MAFF 305635 using pCIH1-mCherry. For the construction of pCIH1-mCherry, ChCIH1 (CH063_13023) including 1012 bp upstream sequences was fused in frame to an mCherry gene in pSL1180 and subsequently cloned into the single PmeI site of binary vector pPN (Korn et al., 2015). Prior to the use in infection assays, fungal strains were grown on oat meal agar plates [5% (w/v) shredded oat meal, 1.2% (w/v) agar] for 7 days at 22°C under illumination to promote conidia formation. Inoculation of leaves from 5-week-old Arabidopsis plants with *Ch* was performed by evenly spraying a conidia suspension at a titer of 2 × 10^6^ conidia/ml onto the leaves as described by Voll et al. (2012). For droplet infections, 5 μl of a 2 × 10^6^ conidia/ml suspension were placed on the leaf surface and plants were further treated as in spray infections.

### Evaluation of Fungal Proliferation and *In Planta* Development

To assess early *in planta* establishment of *Ch* between 1.5 and 2.5 days post infection (dpi), the developmental stages of about 200 to 400 fungal structures were analyzed in whole-leaf mounts after lactophenol-trypan blue staining as described in Koch and Slusarenko (1990) with minor modifications. After boiling freshly sampled leaves in trypan blue staining solution for 1 min, the infected leaves were kept in the staining solution for 24 h and were then transferred to chloral hydrate [250% (w/v)] for destaining on the following day. Microscopy was performed on a Leica DMR microscope (Bensheim, Germany) with DIC optics.

For later infection stages, quantification of the relative genomic DNA content of *Ch* was performed according to Engelsdorf et al. (2013).

### Fluorescence Imaging by Confocal Laser Scanning and Binocular Microscopy

Localization of *At*SWEET11-YFP and *At*SWEET12-YFP in Arabidopsis leaves was performed on a Leica TCS SP5 confocal laser scanning microscope (Leica, Wetzlar, Germany). After excitation with an argon laser at 514 nm, YFP fluorescence was recorded with a photomultiplier tube (PMT) detector between 525 and 560 nm. *Ch* expressing CIH1-mCherry was visualized after excitation with a DPS5 laser at 561 nm and emission PMT detector settings between 570 and 630 nm. Chlorophyll autofluorescence was detected between 657 and 721 nm after excitation with an argon laser.

Localization of YFP signals on the whole leaf scale was performed using a Leica MZ16F binocular (Leica, Wetzlar, Germany) with the corresponding YFP band pass filter set for excitation (510/20 nm) and emission (560/40 nm).

### Determination of Free SA and SAG

Extraction and quantitation of free SA, SAG in leaf samples was performed as described (Voll et al., 2012) with minor modifications reported in Engelsdorf et al. (2013).

### Determination of Soluble Sugar and Starch Contents

Snap-frozen Arabidopsis leaves were extracted twice with 80% ethanol and soluble sugar contents were assayed in a coupled enzymatic assay using a microtiter plate reader as described by Voll et al. (2003). Starch measurements from ethanol insoluble leaf material was performed as described by Voll et al. (2003).

### Transcriptome Analysis by Microarrays

For transcriptome analysis on Agilent Arabidopsis V4 4x44K microarrays (Design-ID 021169, Agilent, Waldbronn, Germany), total RNA was extracted from pools of twelve fully expanded leaves per replicate using the RNase-all method (Chomczynski and Sacchi, 1987). Leaf samples of 5-week-old *sweet11*/*sweet12* and wild type Col-0 plants grown in 12 h light (20°C)/12 h dark cycles (18°C) were either taken immediately before the treatment, at 1 h before the end of the light period (0 dpi) and at 2.5 dpi, at 3 h into the light period. At 2.5 dpi, both mock treated and *Ch* infected samples were taken. Prior to microarray hybridization, RNA quality was assessed by an Agilent 2100 Bioanalyzer (Agilent Technologies, Waldbronn, Germany). Gene expression and GO term enrichment analysis was performed with GeneSpring V12.6 (Agilent Technologies, Waldbronn, Germany).

### Statistical Analysis

Student’s *t*-tests were performed with SigmaPlot 12 (Systat Software Inc., Chicago, IL, United States) after testing for normality (Shapiro–Wilk test) and equal variance.

## Results

### Loss of *AtSWEET11* and *AtSWEET12* Reduces Susceptibility toward *C. higginsianum*

It was shown that SWEET sucrose transporters are induced by bacterial effectors in the phloem of rice leaves to establish compatibility with *Xoo* (Chen et al., 2010; Yuan et al., 2011). In Arabidopsis leaves, various sets of SWEET transporters were found to be induced upon infection with bacterial and fungal pathogens (Chen et al., 2010). Since *At*SWEET11 and *At*SWEET12 were shown to exert redundant functions in phloem loading of Arabidopsis (Chen et al., 2012), we first investigated if either or both transporters might play a role in the interaction of Arabidopsis with *Ch*.

**FIGURE 1:**
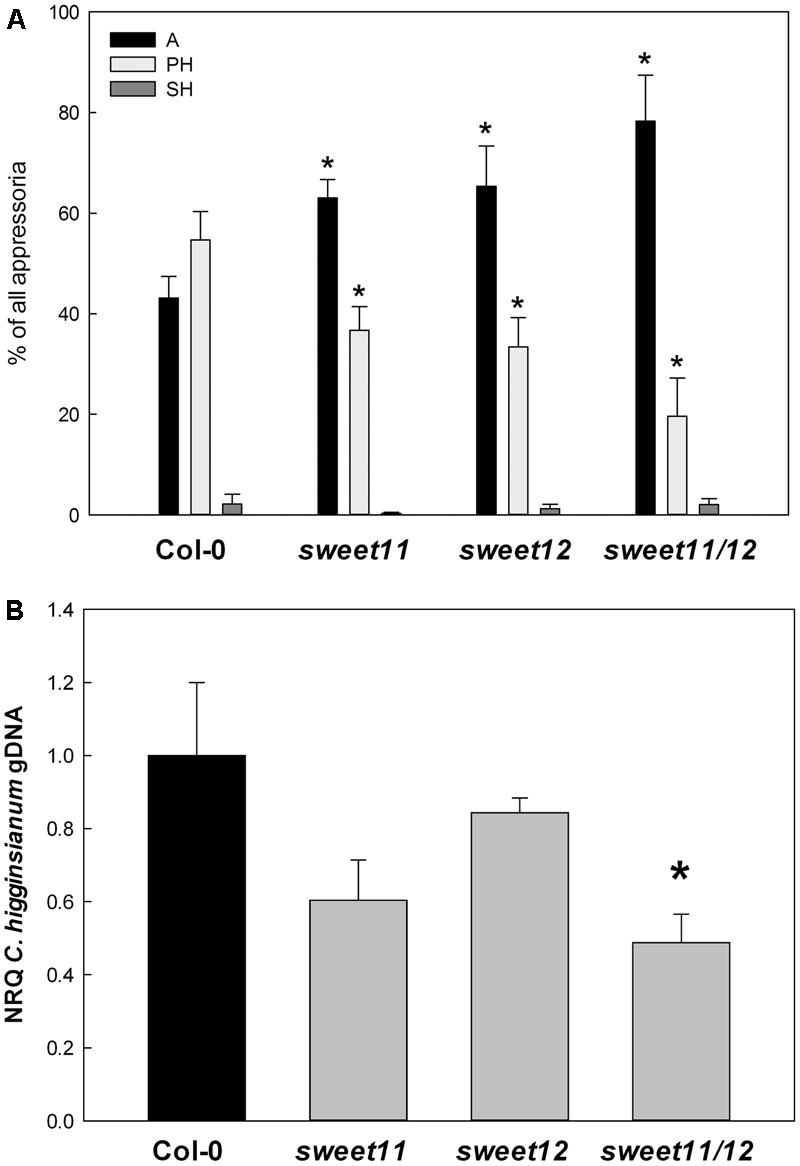
Proliferation of *C. higginsianum* in infected leaves. Plants were grown in 12h /12 h light/dark cycles and five-week old plants were infected at the end of the light period with 2 × 10^6^ *Ch* conidia ml^-1^. **(A)** Early fungal development *in planta* as given by the relative distribution of infection structures. The progress of fungal development was scored in trypan blue stained leaves of the indicated genotypes at 2.5 dpi. Starting from appressoria, the most advanced infection structure derived from each conidium was classified. Per replicate, the developmental status of *in planta* hyphae formed from 200 to 400 conidia was scored. Values are means of four analyzed leaves per genotype with error bars representing the SE. The developmental order is as follows: A, appressoria (black bars), PH, primary hyphae (light gray bars), SH, secondary hyphae (dark gray bars). **(B)** Fungal colonization in the necrotrophic infection phase. As an indicator for *Ch* proliferation, the amount of fungal genomic DNA per leaf area in the indicated genotypes was assessed by qPCR of a genomic *ChTrpC* fragment at 3.5 dpi. Values are means of four independent biological replicates ± SE and represent the normalized relative quantity (NRQ) with Col-0 as a reference. For each replicate, leaf punches from three infected leaves were pooled. Data from one representative out of seven independent replicate experiments with similar results are shown. Asterisks indicate significant differences from Col-0 in a Student’s *t*-test (^∗^*P* < 0.05, ^∗∗^*P* < 0.01, ^∗∗∗^*P* < 0.001).

Immediate post penetration establishment of *Ch* was evaluated microscopically in the middle of the biotrophic phase at 2.5 dpi. The relative number of appressoria (A), biotrophic structures (infection vesicles and primary hyphae, PH) and necrotrophic SH was scored in histological specimen and was taken as a measure for the progress of fungal *in planta* development (**Figure 1A**). In wild type leaves, around 55% of the assessed appressoria had successfully formed biotrophic hyphae *in planta*, while only 20% of the penetration attempts on *sweet11/sweet12* double mutants had resulted in establishment of the biotrophic phase (**Figure 1A**). The *sweet11* and *sweet12* single mutants showed an intermediate phenotype (**Figure 1A**). Necrotrophic hyphae were only occasionally observed at that time point in all genotypes. Thus, the establishment of biotrophy *in planta* was significantly delayed in the *sweet11/sweet12* double mutant, while early post penetration of *Ch* was also attenuated in both single mutants, indicating an additive effect of *AtSWEET11* and *AtSWEET12* deficiency on early post-penetration.

Fungal colonization of the double mutant was reduced by approximately 50% relative to wild type in the early necrotrophic phase at 3.5 dpi (**Figure 1B**), while no significant differences between wild type and both single mutants were observed in any of seven independent replicate experiments (results of one representative experiment are shown in **Figure 1B**).

### *AtSWEET12* Is Locally Induced around *C. higginsianum* Infection Sites

Since deficiency of both transporters showed an additive effect on compatibility, we next assessed if *AtSWEET11* and *AtSWEET12* are induced by *Ch.* Transcript profiling in the biotrophic (2 dpi) and necrotrophic infection phase (4 dpi) indicated that none of the two transporters was substantially induced during biotrophic colonization, while only *AtSWEET12* got strongly induced on the whole leaf scale during the necrotrophic phase of *Ch* infection (**Table 1**).

**Table 1:** Induction of clade III *At*SWEET genes during infection of wild type Col-0 with *C. higginsianum*.

Gene	Gene No.	2 dpi	4 dpi
**Fold change infected vs. control**			
SWEET9	AT2G39060	-2.94	1.12
SWEET10	AT5G50790	n/a	n/a
SWEET11	AT3G48740	1.22	1.70
SWEET12	AT5G23660	1.29	69.66
SWEET13	AT5G50800	1.76	-2.32
SWEET14	AT4G25010	n/a	n/a
SWEET15	AT5G13170	-1.41	1.31

*Transcript data were obtained by microarray analysis of total RNA isolated from fully expanded leaves at the indicated time points after spray infection with 2 × 10^*6*^ conidia/ml. Data were calculated using GeneSpring v12.6, are given as fold change of infected vs. mock treated samples and represent the mean values of three to four biological replicates. n/a – no matching probe set on the Agilent AthV4 microarray.*

**FIGURE 2:**
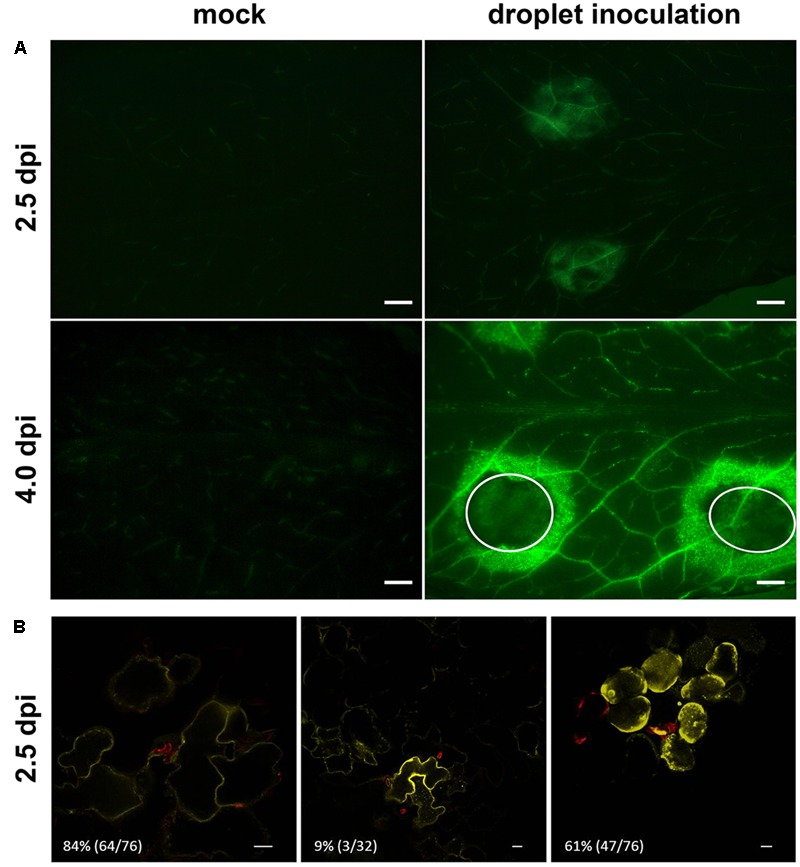
Localization of *pAtSWEET12*:*At*SWEET12-YFP upon *C. higginsianum* infection. **(A)** Analysis of *pAtSWEET12*:*At*SWEET12-YFP accumulation at binocular resolution after droplet inoculation with distilled water in mock control plants (left panel) and with 2 × 10^6^ *Ch* conidia ml^-1^ in infected plants (right panel) at 2.5 dpi (upper row) and 4.0 dpi (lower row). The exposure for each image was 7.3 s, the scale bar indicates 1 mm and white dotted circles highlight necrotic regions. Please note that YFP localization was recorded in the long-wave GFP channel. **(B)** Local induction of *pAtSWEET12*:*At*SWEET12-YFP in the biotrophic interaction phase at 2.5 dpi in neighboring epidermis cells (left), infected cells themselves (middle) and mesophyll cells below the infected cells (right) using CLSM. Five week-old plants were spray-infected with a *Ch* strain expressing CIH1-mCherry at a titer of 2 × 10^6^ conidia ml^-1^. Images represent overlays of *At*SWEET12-YFP (yellow channel) and CIH-mCherry (red channel) fluorescence. Please note that for the sake of clarity, the overlay for mesophyll cells (right panel) is composed of an YFP image from the mesophyll focus plane and an mCherry image from the epidermal plane directly above. Numbers in the bottom left of each image indicate the relative incidence of the respective induction pattern per total investigated specimen in percent. The scale bar represents 20 μm. Data from one representative out of five independent replicate experiments with similar results are shown.

To assess the local and temporal induction of *AtSWEET11* and *AtSWEET12* more closely, we performed droplet inoculation with *Ch* on *pAtSWEET11*:*At*SWEET11-YFP and *pAtSWEET12*:*At*SWEET12-YFP reporter plants. Confirming the microarray data, *At*SWEET11-YFP protein was detected in vascular tissue of infected and mock control leaves, but no further induction of *At*SWEET11-YFP was observed in *Ch* infected leaves (Supplementary Figure S1A). In contrast, *At*SWEET12-YFP accumulated at *Ch* infection sites during the biotrophic phase at 2.5 dpi, and strong expression of *At*SWEET12-YFP fusion protein was detected around necrotic lesions during the necrotrophic infection phase at 4 dpi (**Figure 2A**, see Supplementary Figure S1B for images from an independent *pAtSWEET12*:*At*SWEET12-YFP reporter line). In addition, *At*SWEET12-YFP accumulation was much stronger in the vasculature of infected compared to control plants at both stages of the infection (**Figure 2A**). To assess the local induction of *AtSWEET12* during the biotrophic phase at the cellular level, we infected the *pAtSWEET12*:*At*SWEET12-YFP reporter lines with a *Ch* strain expressing a secreted LysM-Domain CIH1-mCherry reporter protein, which accumulates around primary hyphae *in planta* (**Figure 2B** and Supplementary Figure S2). Interestingly, we never detected *At*SWEET12-YFP around primary hyphae of the pathogen by CLSM. An accumulation of *At*SWEET12-YFP was only observed in 9% of the penetrated epidermis cells (**Figure 2B**). By contrast, neighboring epidermis cells and underlying mesophyll cells showed *At*SWEET12-YFP reporter fluorescence in 84 and 61% of all cases, respectively (**Figure 2B**).

The absence of *At*SWEET11-YFP and *At*SWEET12-YFP from the interfacial matrix strongly argues against a role of these two transporters in providing sugars to *Ch* directly at the plant–pathogen interface. However, induction of *At*SWEET12 in mesophyll and epidermis cells surrounding the infected cell might enrich the apoplasmic compartment at the infection site with sucrose. Therefore, we investigated major carbohydrate metabolism in control and *Ch* infected leaves in more detail.

### *sweet11/12* Double Mutant Leaves Show Elevated Sugar Turnover and Accumulate Hexoses

Since the *sweet11/sweet12* double mutant exhibited an approximately 50% reduction in sugar exudation and reduced nocturnal starch mobilization (Chen et al., 2012), we expected that soluble sugar levels are constantly elevated in the *sweet11/sweet12* double mutant.

**FIGURE 3:**
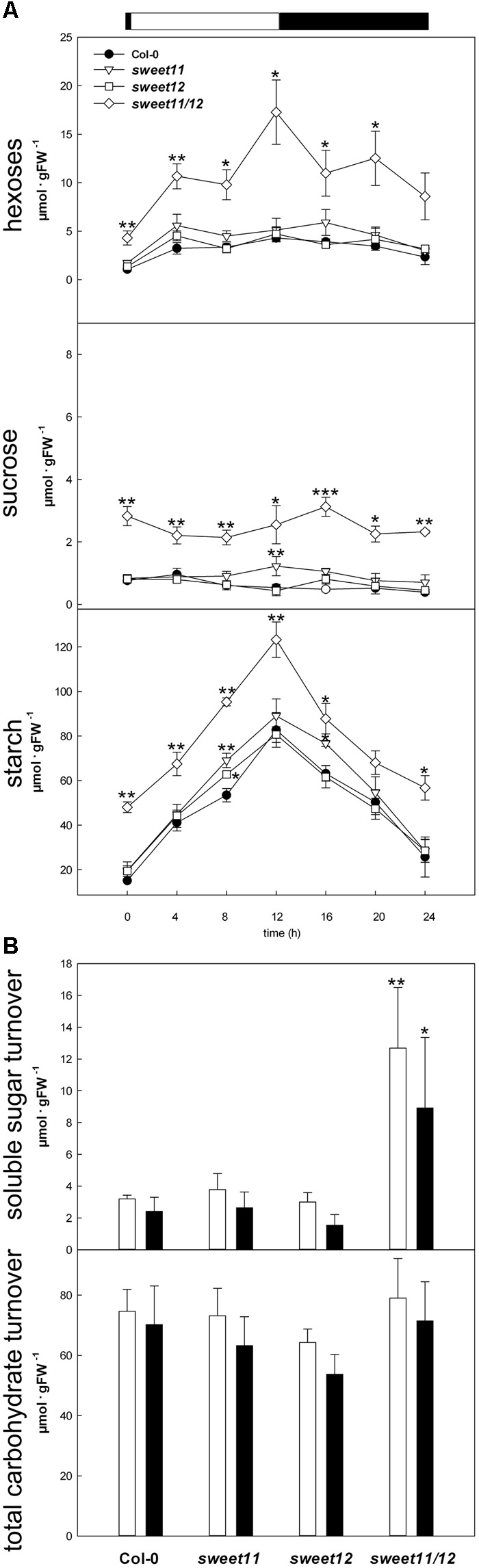
Time course of soluble sugar and starch contents and diurnal carbohydrate turnover. **(A)** Contents of hexoses (top panel), sucrose (middle panel) and starch (bottom panel) in untreated leaves of Col-0 (black circles), *sweet11* (white triangles), *sweet12* (white squares) and *sweet11/sweet12* (white diamonds) in a 12 h/12 h light/dark cycle. Data for short and long day can be found in Supplementary Figure S3. The bar above the top panel indicates the light (white) and the dark phase (black). **(B)** Diurnal turnover of soluble sugars (top panel) and total carbohydrate content (bottom panel), as calculated from the data depicted in **(A)**. Carbohydrate accumulation, white bars; carbohydrate mobilization, black bars. Values are means of five biological replicates ± SE. Asterisks indicate significant differences from Col-0 in a Student’s *t*-test (^∗^*P* < 0.05, ^∗∗^*P* < 0.01, ^∗∗∗^*P* < 0.001). Data from one representative out of two independent replicate experiments with similar results are shown. FW, Fresh weight.

To study major carbohydrate contents in more detail, we measured the contents of soluble sugars and starch in leaves during diurnal light/dark cycles in short day (8 h light/16 h dark), 12 h light/12 h dark cycles and long day conditions (16 h light/8 h dark). In all light regimes, the *sweet11/sweet12* double mutant showed strongly elevated contents of hexoses, sucrose and starch throughout the diurnal cycle compared to wild type and the single mutants (**Figure 3A** and Supplementary Figure S3). Throughout the diurnal cycle, sucrose and starch contents remained constantly elevated in the double mutants compared to the other genotypes, while hexose accumulated in the double mutant during the first half of the light period (**Figure 3A** and Supplementary Figure S3). To assess more closely, if the rate of starch and soluble sugar accumulation (i.e., the content at the end of light minus the content at the end of the preceding dark period) as well as starch and soluble sugar mobilization (i.e., the content at the end of light minus the content at the end of the following dark period) differ between the genotypes, we calculated the turnover of total soluble sugars and starch from the time course data (**Figure 3B** and Supplementary Figure S3). While the turnover of soluble sugars was significantly increased in the *sweet11/sweet12* double mutant under all studied diurnal cycles, the calculated total diurnal carbohydrate accumulation and mobilization were comparable between all other genotypes (**Figure 3B** and Supplementary Figure S3).

**FIGURE 4:**
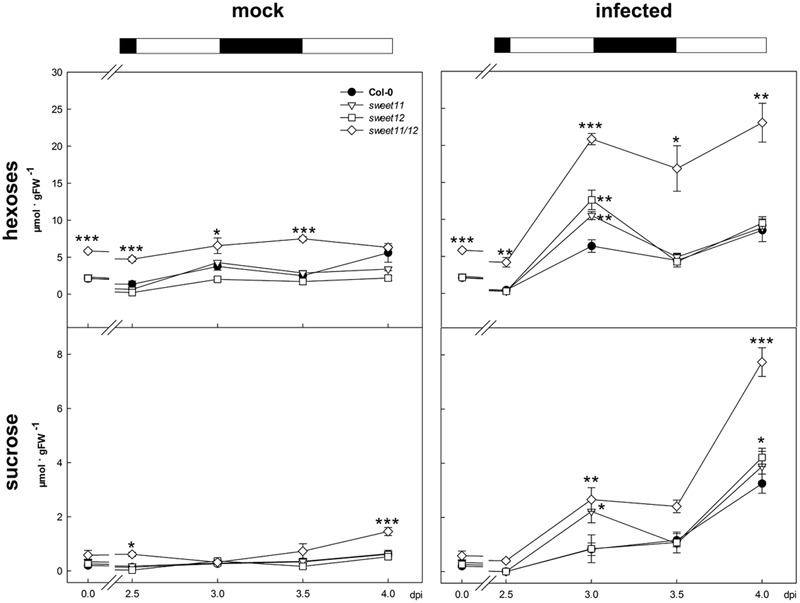
Accumulation of soluble sugars in *C. higginsianum* infected leaves. The contents of soluble sugars of untreated (left) and *Ch* infected plants (right), grown in a 12 h/12 h light/dark rhythm were monitored at the end of the light (0, 3.0, and 4.0 dpi) and at the end of the dark period (2.5 and 3.5 dpi). Col-0, black circles; *sweet11*, white triangles; *sweet12*, white squares; *sweet11/12*, white diamonds. Values are means of five biological replicates ± SE. Asterisks indicate significant differences from Col-0 in a Student’s *t*-test (^∗^*P* < 0.05, ^∗∗^*P* < 0.01, ^∗∗∗^*P* < 0.001). Data from one representative out of two independent replicate experiments with similar results are shown. FW, Fresh weight.

Infected leaves of all genotypes accumulated more hexoses and sucrose than the corresponding mock controls at the end of the light periods at 3 and 4 dpi. Hexose contents in *Ch* infected leaves were twice as high in the double mutant as compared to the other genotypes at all time points, while sucrose contents were elevated in infected double mutant compared to infected wild type leaves at the end of the subjective light phases at 3 and 4 dpi (**Figure 4**). Taken together, the progressive accumulation of hexoses and sucrose in the course of *Ch* infection was much more pronounced in double mutant leaves compared to wild type and single mutants. Leaves of infected single mutants only exhibited a transient increase of hexose and sucrose contents compared to infected wild type leaves at 3 dpi (**Figure 4**).

### Increased Resistance of *sweet11/sweet12* toward *C. higginsianum* Depends on Salicylic Acid (SA)

**FIGURE 5:**
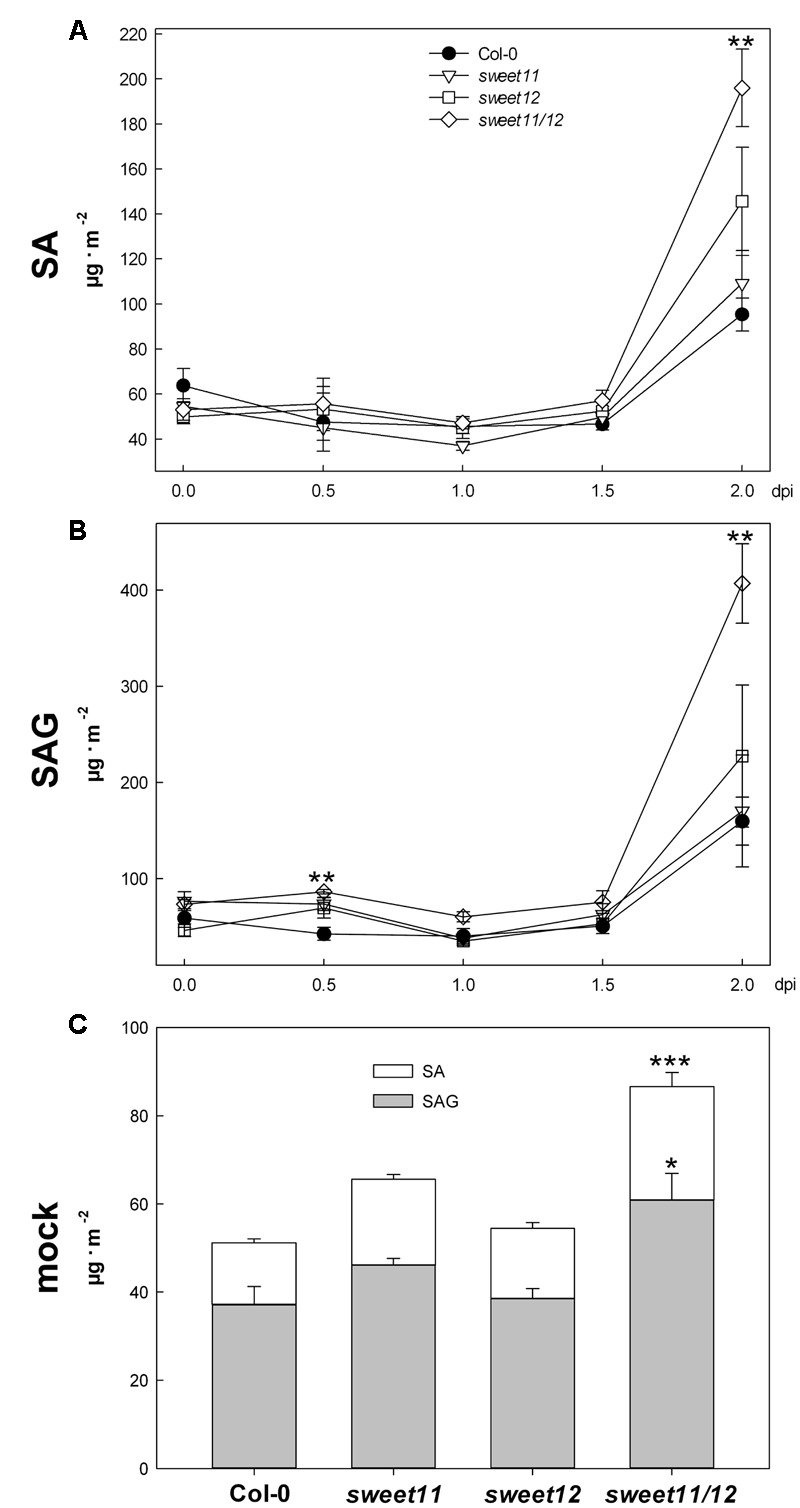
Free SA and SAG during the early biotrophic interaction phase of Arabidopsis and *C. higginsianum*. Plants grown in 12 h/12 h light/dark cycles were infected with *Ch* at the end of the light period and contents of SA **(A)** and SAG **(B)** were measured in 12 h intervals at the end of the light period (0, 1.0 dpi, and 2.0 dpi) and at the beginning of the light period (0.5 and 1.5 dpi). Col-0, black circles; *sweet11*, white triangles; *sweet12*, white squares; *sweet11/12*, white diamonds. **(C)** SA (white bars) and SAG (gray bars) contents of mock treated plants at 2.5 dpi. Values are means of five biological replicates ± SE. Asterisks indicate significant differences from Col-0 (^∗^*P* < 0.05, ^∗∗^*P* < 0.01, ^∗∗∗^*P* < 0.001, Student’s *t*-test). Data from one representative out of two independent replicate experiments with similar results are shown.

The observed elevated levels of soluble sugars, especially hexoses, in mock treated *sweet11*/*sweet12* double mutants – as well as the enhanced accumulation of soluble sugars upon challenge with *Ch* – may elicit sugar mediated priming of the SA pathway, as described by Linke et al. (2002) and Conrath et al. (2006). Therefore, we monitored contents of free SA as well as conjugated SA glucoside (SAG) in leaves of untreated control plants, mock treated plants and *Ch* infected plants in 12 h intervals from the time of treatment until 2.5 dpi. At all time points, SA and SAG contents in untreated leaves ranged between 40 and 50 μg⋅m^-2^ and 60 and 80 μg⋅m^-2^, respectively, and did not differ significantly between genotypes. Both SA and SAG contents rapidly increased at the time of biotrophic establishment of *Ch* at 2 dpi in all genotypes (**Figures 5A,B**). While the timing of SA production was comparable among all genotypes, SA and SAG accumulation was increased approximately twofold in *sweet11*/*sweet12* compared to wild type at 2 dpi (**Figure 5**).

Interestingly, the contents of free SA and SAG were also significantly elevated by around twofold in mock treated *sweet11*/*sweet12* double mutants compared to mock treated wild type, i.e., in plants that were sprayed with water and kept at high humidity (**Figure 5C**). This might be connected to sugar-mediated priming of the SA pathway in the absence of pathogen. We employed microarray analysis to investigate the response of SA regulated genes in mock treated leaves at 2.5 dpi. In mock treated double mutants, 645 genes were significantly down-regulated and 345 genes were significantly up-regulated compared to mock treated wild type at 2.5 days post treatment (Supplementary Table S1). More than 13% of those genes that were significantly induced in *sweet11*/*sweet12* double mutants, i.e., 134 genes, were associated with plant defense responses according to GO term annotation. GO categories associated with defense, incompatible interactions and SA associated pathways were highly significantly enriched in this gene set (**Table 2**), corroborating that mock treated *sweet11*/*sweet12* leaves are in a primed state. Concomitantly, *Ch* infected double mutant leaves exhibited a significant enrichment of the antagonistic JA response among the down-regulated genes compared to infected wild type leaves at 2.5 dpi (corrected *p*-value 2.57 × 10^-5^), indicating a faster establishment of the SA response in *sweet11*/*sweet12* upon *Ch* challenge. Most obviously, dozens of GO terms associated with photosynthetic and primary metabolism were significantly enriched among the genes induced in *Ch* infected double mutant leaves compared to *Ch* infected wild type (Supplementary Table S2), reflecting a reduced degree of infection and, consequently, increased metabolic capacity of the double mutant at 2.5 dpi. In untreated *sweet11*/*sweet12* double mutants only 114 genes were significantly up- and down-regulated, respectively, compared to untreated Col-0 wild type (Supplementary Table S3). An enrichment of defense associated GO categories was absent from these gene sets.

**Table 2:** GO term enrichment analysis of genes induced in mock treated leaves of *sweet11*/ *sweet12* double mutants.

GO term	Corrected *p*-value
Defense response	1.26 × 10^-26^
Defense response, incompatible interaction	8.30 × 10^-25^
SAR	1.26 × 10^-18^
SA biosynthetic process	1.14 × 10^-16^
Defense response fungus	4.87 × 10^-13^
MAPK cascade	6.80 × 10^-12^
Regulation of immune response	1.25 × 10^-9^
Regulation of ROS metabolic process	1.46 × 10^-9^
Defense response to bacterium	1.87 × 10^-8^

*Transcript data from three independent biological replicates were obtained by microarray analysis of total RNA isolated from fully expanded leaves at 2.5 days after the onset of mock treatment, i.e., high humidity, as described in the materials and methods section. GO term enrichment analysis of genes differentially expressed with a fold change >2 and *p* < 0.05 in *sweet11*/*sweet12* double mutant vs. wild type Col-0 samples was conducted with GeneSpring v12.6. Significantly enriched GO terms are in ascending order based on corrected *p*-values.*

Taken together, *sweet11*/*sweet12* double mutants exhibit enhanced SA accumulation and an induction of SA regulated genes already in mock treated leaves. Since SA mediated responses are reportedly very effective in defense of *Ch* during biotrophy (O’Connell et al., 2004; Birker et al., 2009), a faster induction of the SA triggered defense response might explain the reduced susceptibility of the double mutant toward *Ch*. To provide genetic evidence for the role of the SA priming for the diminished susceptibility of double mutants toward *Ch*, we investigated fungal colonization in *sweet11*/*sweet12/sid2* triple mutants, which lack the committed step of SA biosynthesis, isochorismate synthase, and hence, SA (Wildermuth et al., 2001).

**FIGURE 6:**
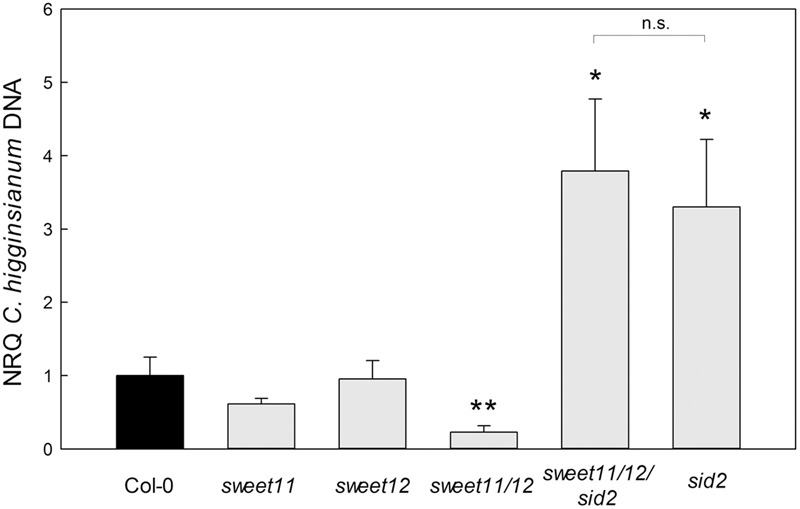
Diminished susceptibility of *sweet11/sweet12* toward *C. higginsianum* depends on SA.Fungal colonization during the early necrotrophic phase at 3.5 dpi was assessed as the amount of fungal genomic DNA per leaf area of the indicated genotypes by qPCR, as described in the legend of **Figure 1B**. Values are means of five independent biological replicates ± SE and represent the normalized relative quantity (NRQ) with Col-0 as a reference. For each replicate, leaf punches from three infected leaves were pooled. Asterisks indicate significant differences from Col-0 in a Student’s *t*-test (^∗^*P* < 0.05, ^∗∗^*P* < 0.01, ^∗∗∗^*P* < 0.001).

Fungal colonization of wild type Col-0, *sweet11, sweet12*, and *sweet11/sweet12* mutants at 3.5 dpi was comparable to the data shown in **Figure 1**, while the SA deficient mutant *sid2* was hypersusceptible toward *Ch* (**Figure 6**). Intriguingly, the *sweet11*/*sweet12/sid2* triple mutant shared the hypersusceptible phenotype with *sid2*, demonstrating that the diminished susceptibility of the *sweet11/sweet12* mutant depends on the SA pathway.

## Discussion

### *AtSWEET11* and *AtSWEET12* Do Not Play a Major Role in the Interaction of Arabidopsis with *C. higginsianum*

Besides SWEET transporters that have been identified as direct targets of bacterial TAL effectors in rice and cassava (Chu et al., 2006; Yang et al., 2006; Antony et al., 2010; Chen et al., 2010; Liu et al., 2011; Yu et al., 2011; Cohn et al., 2014), a strong transcriptional induction of several *SWEET* transporters has been reported during the interaction of Arabidopsis and grapevine with systematically diverse biotrophic and necrotrophic pathogens (Chen et al., 2010; Chong et al., 2014), which may indicate that reprogramming of *SWEET* transporters for the diversion of organic carbon from the host tissue is a recurring motif during microbial attack. Based on the previously observed transcriptional induction seen by Chen et al. (2010), our study was set out to find indication, if reprogramming of the phloem localized Arabidopsis transporters *At*SWEET11 and *At*SWEET12 might be vital for carbon supply to *C. higginsianum*.

We observed that early post penetration establishment during the biotrophic phase of *Ch* was delayed in both, *sweet11* and *sweet12* single mutants and an additive effect of both mutations in the double mutant was evident. This may indicate a role of *At*SWEET11 and *At*SWEET12 in nutrient supply to *Ch*, but it is impossible to determine *in vivo* carbon transfer rates that could provide direct experimental evidence to verify this hypothesis.

Nevertheless, circumstantial evidence speaks against this hypothesis. While both single mutants showed delayed establishment of *Ch* in the early biotrophic phase at 2 dpi, only *At*SWEET12-YFP reporter protein accumulated around *Ch* infection sites. Fungal proliferation in the *sweet12* single mutant remained unaffected at the end of the biotrophic phase at 3.5 dpi, demonstrating that *At*SWEET12 accumulation around the infection site cannot have a substantial influence on the interaction. In contrast, *AtS*WEET11-YFP expression remained unaffected in *Ch* infected leaves and was restricted to vascular tissue, making it very unlikely that *AtS*WEET11 confers major changes in carbohydrate allocation in *Ch* infected leaves. If *AtSWEET11* or *AtSWEET12* were targets of fungal effector proteins and were reprogrammed for diversion of assimilates from colonized leaf tissue, a strong induction of the two SWEET fusion proteins at the plant–fungal interface, i.e., the interfacial matrix, around biotrophic hyphae of *Ch* would have been expected. This was clearly not the case. Recent data indicate that the initial so-called biotrophic phase of *Ch* rather serves defense suppression than nutrient uptake (O’Connell et al., 2012; Engelsdorf et al., 2013). None of the 93 potential sugar transporters encoded in the *Ch* genome were highly induced in biotropic hyphae *in planta* and only 13 sugar transporters were exclusively transcribed in biotrophic hyphae (O’Connell et al., 2012). In addition, *C. higginsianum* was found to be much more virulent on plants that had been kept in the dark for 60 h and were devoid of free sugars (Engelsdorf et al., 2013). In summary, it is to be expected that the pathogen is largely independent of host sugar transporter expression, if *Ch* does not rely on carbon provision by host cells during the initial biotrophic phase.

### Diminished Susceptibility of *sweet11/sweet12 Double Mutants* toward *C. higginsianum* Can Be Explained by Sugar-Mediated Defense Priming

The *sweet11*/*sweet12* double mutant exhibited reduced susceptibility toward *Ch* both during the biotrophic and in the ensuing necrotrophic colonization phase, which may indicate that *AtSWEET11* and *AtSWEET12* might act redundantly for the nutrition of *Ch*. While *AtSWEET11* expression could never be detected outside the vasculature, *AtSWEET12* was strongly induced in vicinity of *Ch* infection sites. This argues against a functional redundancy of *At*SWEET11 and *At*SWEET12 on the local scale at the interaction site, also excluding a major role of the two transporters in carbon provision to the fungal pathogen.

However, both transporters were expressed in the vasculature of *Ch* infected leaves. Therefore, it seems reasonable to associate the functional redundancy of *At*SWEET11 and *At*SWEET12 during *Ch* challenge with their role in phloem loading (Chen et al., 2012). Unlike *sweet11* and *sweet12* single mutants, the *sweet11/sweet12* double mutant exhibited elevated steady state contents of hexoses, sucrose and starch throughout the diurnal cycle, irrespective of the duration of the light phase. This physiological phenotype indicates that *At*SWEET11 and *At*SWEET12 play redundant roles for major leaf carbohydrate metabolism. Furthermore, *At*SWEET11 and *At*SWEET12 appear to have an additive effect on compatibility toward *Ch* (see data in **Figure 1**). If we assume that their impact on compatibility is connected to their function in carbohydrate export, it seems reasonable that the loss of *At*SWEET11 has a more pronounced effect on compatibility than *At*SWEET12, since the expression level of *At*SWEET11 is more than twofold higher than that of *At*SWEET12 (as analyzed with the eFP browser^1^).

It has previously been demonstrated that reduced carbohydrate availability in Arabidopsis leaves correlates with susceptibility toward *Ch* (Engelsdorf et al., 2013). Furthermore, reduced availability of carbohydrates hampered the induction of SA triggered PR genes (Engelsdorf et al., 2013). In turn, diminished susceptibility of the *sweet11/sweet12* double mutant toward *Ch* may simply be explained by a surplus of carbohydrates compared to the other genotypes. Carbohydrates that fail to be exported from the symplast into the apoplasm will likely accumulate in the cytosol (and in the vacuole) of *sweet11/sweet12* leaf cells. It has recently been demonstrated that the PAMP-triggered activation of the hexose transporter STP13 reduces compatibility toward *Pseudomonas syringae* pv. *tomato* by influencing the allocation of sugars in expense of the apoplasm and in favor of the host cytosol (Yamada et al., 2016). Likewise, these extra carbon reserves in the *sweet11/sweet12* double mutant might allow for increased metabolic activity, and hence an enhanced defense response. In support of the proposed connection between elevated carbon status and enhanced defense in the double mutant, we have observed an accumulation of free SA, SA-glucosides (SAG) as well as a highly significant induction of genes involved in the SA mediated defense response not only in *Ch* infected, but also in mock treated *sweet11*/*sweet12* double mutants relative to wild type. Interestingly, the described stimulation of the SA pathway was absent from untreated double mutants, indicating that elevated contents of soluble sugars are not sufficient to evoke a response of the SA pathway in *sweet11/sweet12* in general. Our observations suggest that the stimulation of SA accumulation can either be triggered upon challenge with *Ch*, or may also occur in the presence of abiotic stimuli like high humidity, as in mock treated plants.

Priming is defined as ‘a physiological state in which plants are able to faster and better activate defense responses’ (Beckers and Conrath, 2007; Moghaddam and Van den Ende, 2012). Defense priming can either be achieved by contact to avirulent pathogens or symbiotic microbes, by particular physiological conditions, or by chemical treatment (for an overview, please see Beckers and Conrath, 2007). While systemic acquired resistance (SAR) upon pathogen challenge is mediated by pipecolic acid (Bernsdorff et al., 2016), it has been shown that mobilization of SAG into free SA plays a key role in chemical priming of the SA response (Noutoshi et al., 2012). Consistently, the pool size of SAGs was 3.5-fold elevated in primed *sweet11/sweet12* compared to Col-0 wild type in our study, while the difference in SAG pool size was less than 1.5-fold between double mutants and wild type in control conditions and after *Ch* challenge. Since a positive influence of sugar metabolism on the SA pathway is well-known (as compiled by Bolton, 2009), we assume that constantly elevated levels of soluble sugars, especially hexoses, promote defense priming in *sweet11*/*sweet12*. A stimulation of SA-dependent PR gene expression in pathogen or elicitor challenged, but not in untreated, potato tubers with antisense suppression of the plastidic ATP/ADP transporter AATP1 has previously been observed by Linke et al. (2002). Interestingly, AATP1 antisense tubers exhibited an elevated energy charge and 10-fold increased contents of glucose compared to controls (Tjaden et al., 1998). The increase in hexose content in leaves of *sweet11/sweet12* double mutants was substantial throughout the diurnal cycle, but only 3.5-fold elevated compared to wild type. Nevertheless, it seems reasonable to assume a similar physiological scenario in *sweet11/sweet12* double mutant leaves compared to AATP1 potato tubers. Furthermore, it has been demonstrated that priming depends on MAP kinase signaling via MPK3 and MPK6 (Beckers et al., 2009). In turn, MAPK signaling belonged to the six most significantly enriched GO terms of genes induced in mock treated *sweet11/sweet12* double mutants compared to wild type, providing further evidence for priming of the SA pathway in *sweet11/sweet12*. The observation that the effect of the *sid2* mutation on compatibility is epistatic over *sweet11*/*sweet12* in the *sweet11*/*sweet12/sid2* triple mutant unequivocally demonstrates that diminished susceptibility in *sweet11*/*sweet12* is conferred by SA accumulation and the SA pathway. It is known since more than a decade that the SA triggered defense response is very effective against *Ch* infection during the initial biotrophic phase (Narusaka et al., 2004; O’Connell et al., 2004).

## Conclusion

Our results indicate that diminished susceptibility of *sweet11*/*sweet12* double mutants toward *Ch* is not caused by impaired sugar provision to the pathogen, but by the sugar-primed activation of the SA pathway.

## Author Contributions

US, CK, and LV conceived the project. MK produced all the fungal strains used and PG, MK, and TE performed the described experiments. All authors analyzed the data. LV and PG wrote the manuscript. US and CK edited the manuscript.

## Conflict of Interest Statement

The authors declare that the research was conducted in the absence of any commercial or financial relationships that could be construed as a potential conflict of interest.

## Footnotes

1 http://bar.utoronto.ca/efp/cgi-bin/efpWeb.cgi
