# Supplementary material for: Sugar Accumulation in Leaves of Arabidopsis sweet11/sweet12 Double Mutants Enhances Priming of the Salicylic Acid-Mediated Defense Response
Source: Front Plant Sci. 2017 Aug 8;8:1378. doi: 10.3389/fpls.2017.01378 (PMC5550771; doi:10.3389/fpls.2017.01378)
Supplement: Supplementary file 4 [file Data_Sheet_1.docx]

**Supplementary Data – Priming causes fungal resistance**

**Supplementary Figures**


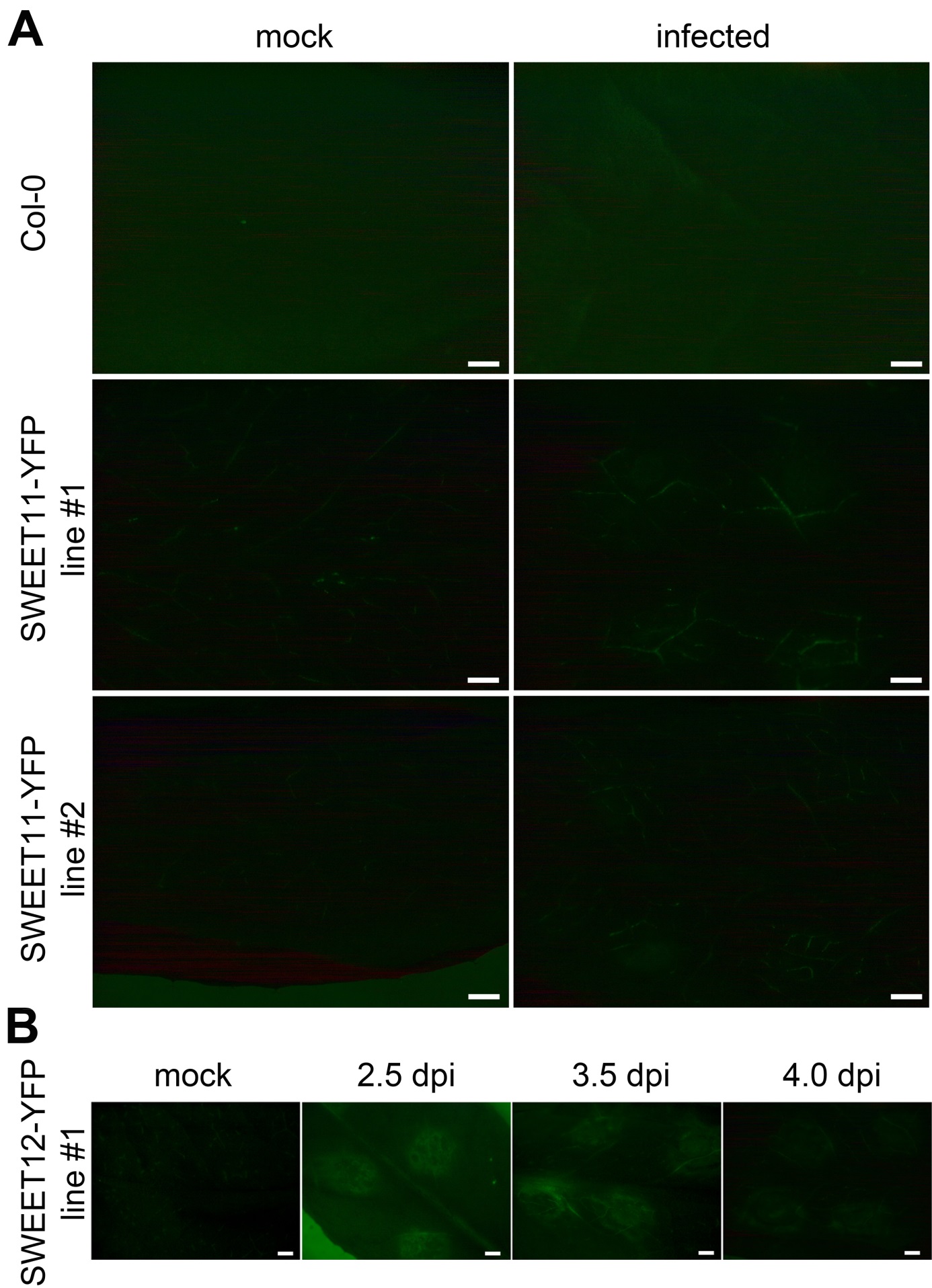


**Figure S1. *C. higginsianum* droplet infection on *pAtSWEET11*:*At*SWEET11-YFP and an independent *pAtSWEET12*::*At*SWEET12-YFP line.** Five week old plants were cultivated in 12-h/12-h light/dark cycles and were infected at the end of the light period with three to four droplets of 5 µl per leaf containing 2 x 10^6^ conidia ml^-1^ of *C. higginsianum*. **A** Analysis of *At*SWEET11-YFP by binocular after droplet inoculation of water control plants (left panel) and infected plants (right panel) at 4.0 dpi. The exposure time was 7.3 seconds and the scale bar indicates 1 mm. YFP localization was recorded in the long-wave GFP channel. **B** Investigation of increased *At*SWEET12-YFP expression surrounding the droplet infected area at 2.5 dpi, 3.5 dpi and 4.0 dpi. Scale bar 1 mm. Please note that YFP localization was recorded in the long-wave GFP channel.


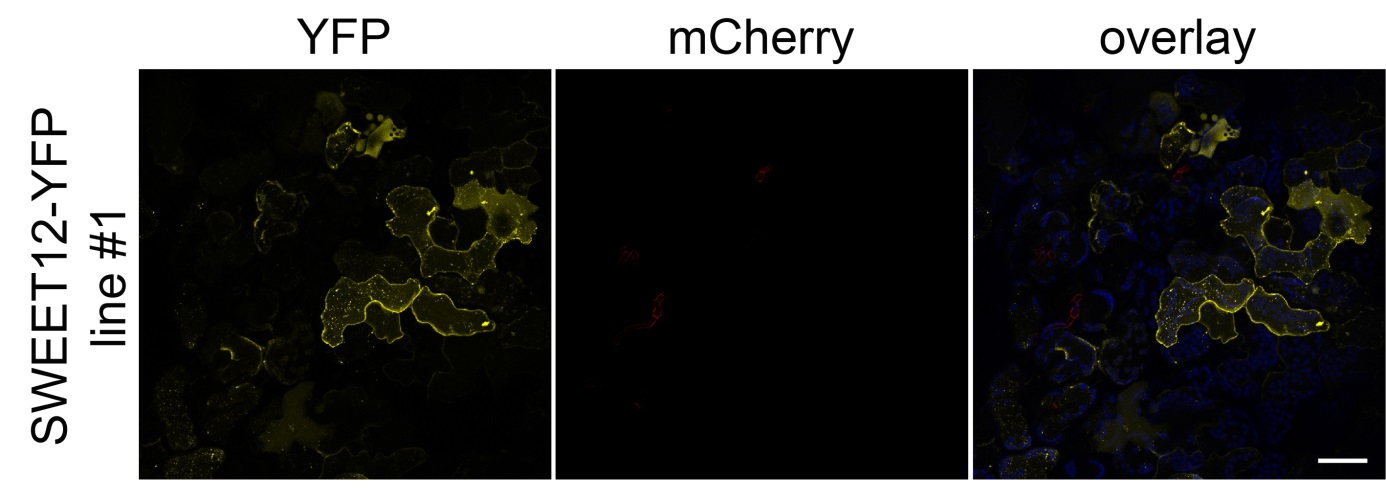


**Figure S2. Localization of *pAtSWEET12*::*At*SWEET12-YFP in an independent line at 2.5 days post *C. higginsianum* infection.** Confocal images of AtSWEET12‑YFP plants at 2.5 days after spray inoculation with a *C. higginsianum* strain expressing CIH1-mCherry. The used fluorescence channels, YFP (left), mCherry (middle) and overlay (right) are indicated above the pictures. Scale bar 50 µm.


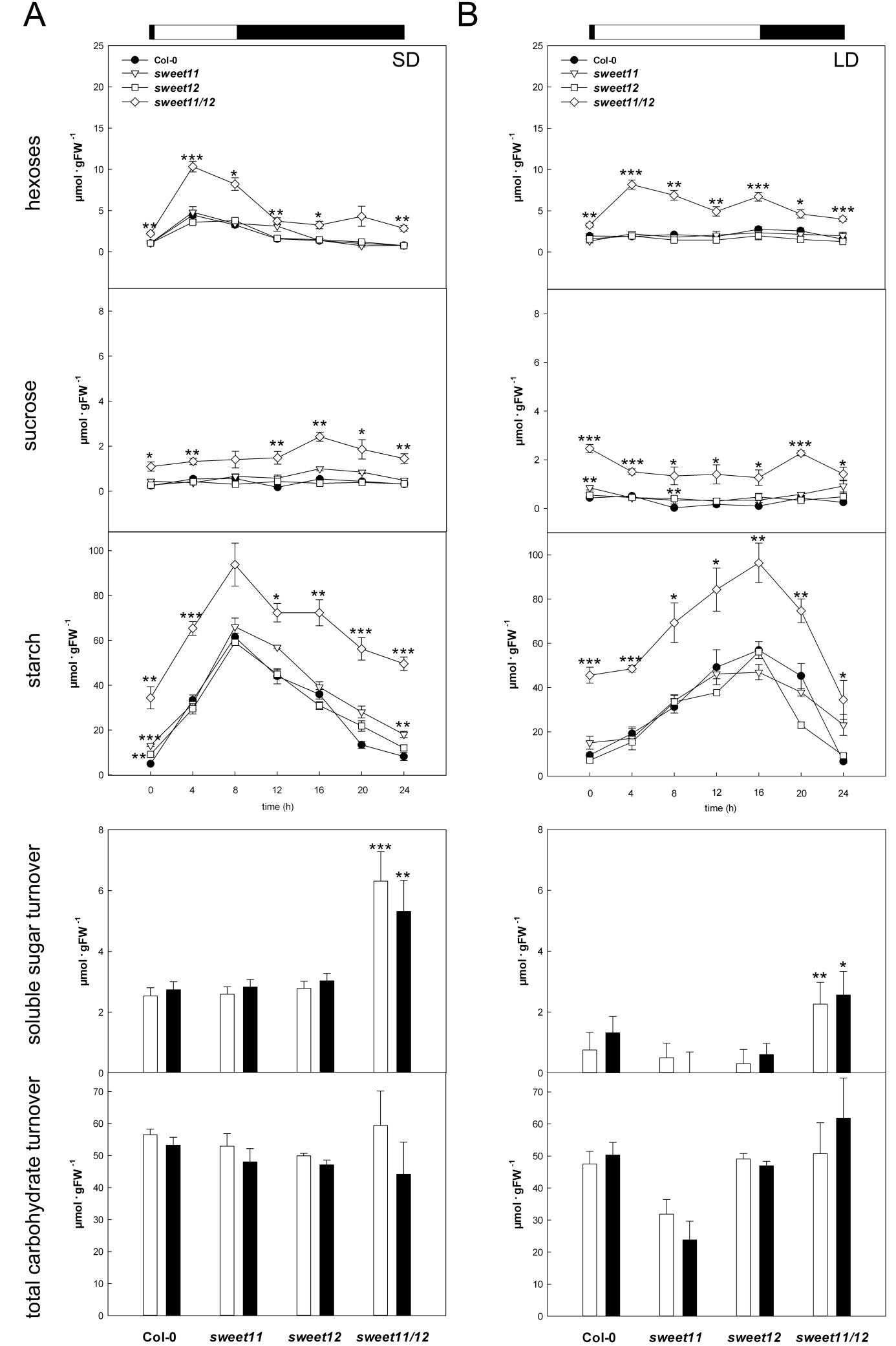


**Figure S3. Diurnal variation of soluble sugar and starch contents and diurnal carbohydrate turnover in short day (A, left) and long day conditions (B, right).** The bar above the top panel indicates the light (white) and the dark phase (black). Contents of hexoses (top row), sucrose (second row) and starch (third row) in untreated leaves of Col-0 (black circles), *sweet11* (white triangles), *sweet12* (white squares) and *sweet11/sweet12* (white diamonds) are depicted. Diurnal turnover of soluble sugars (fourth row) and total carbohydrate content (bottom panel) were calculated from the data depicted in rows 1 to 3. Carbohydrate accumulation, white bars; carbohydrate mobilization, black bars. Values are means of five biological replicates ± SE. Asterisks indicate significant differences from Col-0 in a Student´s *t*‑test (**P* < 0.05, ***P* < 0.01, ****P* < 0.001). FW, Fresh weight.

**Supplementary Tables**

**Table S1. Differentially regulated genes between *sweet11/sweet12* double mutant and Col-0 wild type leaves after 2.5 days of mock treatment.**

Agilent Arabidopsis V4 4x44K microarray raw data were normalized and baseline transformed to the 75^th^ percentile. Differentially regulated genes between the double mutant and wild type with a fold change > 2 and p-value < 0.05 were determined by a Volcano Plot.

Microarray data obtained in this study which is deposited in the Gene Expression Omnibus (<http://www.ncbi.nlm.nih.gov/geo/>) under the accession number GSE 67544 and reviewer access is available upon request.

**Table S2. GO term enrichment analysis of genes induced in *Ch* infected leaves of *sweet11*/ *sweet12* double mutants compared to Col-0 wild type.**

Transcript data from three independent biological replicates were obtained by microarray analysis of total RNA isolated from fully expanded leaves at 2.5 days after inoculation with *C. higginsianum* as described in the materials and methods section. GO term enrichment analysis of genes differentially expressed with a fold change >2 and p<0.05 in *sweet11*/*sweet12* double mutant vs. wild type Col-0 samples was conducted with GeneSpring v12.6. Significantly enriched GO terms are in ascending order according to corrected p-values.

**Table S3. List of differentially regulated genes between untreated *sweet11/sweet12* double mutant and Col-0 wild type leaves.**

Agilent Arabidopsis V4 4x44K microarray raw data were normalized and baseline transformed to the 75^th^ percentile. Differentially regulated genes between the double mutant and wild type with a fold change > 2 and p-value < 0.05 were determined by a Volcano Plot.

Microarray data obtained in this study which is deposited in the Gene Expression Omnibus (<http://www.ncbi.nlm.nih.gov/geo/>) under the accession number GSE 67544 and reviewer access is available upon request.
